# A novel oncolytic virus induces a regional cytokine storm and safely eliminates malignant ascites of colon cancer

**DOI:** 10.1002/cam4.4772

**Published:** 2022-05-05

**Authors:** Shuang Dong, Binlei Liu, Sheng Hu, Fang Guo, Yi Zhong, Qian Cai, Siqi Zhang, Yu Qian, Jun Wang, Fuxiang Zhou

**Affiliations:** ^1^ Hubei Key Laboratory of Tumor Biological Behaviors, Department of Radiation and Medical Oncology Zhongnan Hospital of Wuhan University Wuhan Hubei China; ^2^ Department of Oncology, Hubei Cancer Hospital, Tongji Medical College Huazhong University of Science and Technology Wuhan Hubei China; ^3^ National “111” Center for Cellular Regulation and Molecular Pharmaceutics, Key Laboratory of Fermentation Engineering (Ministry of Education), Hubei Provincial Cooperative Innovation Center of Industrial Fermentation, Hubei Key Laboratory of Industrial Microbiology Hubei University of Technology Wuhan Hubei China; ^4^ Department of Pathology, Hubei Cancer Hospital, Tongji Medical College Huazhong University of Science and Technology Wuhan Hubei China; ^5^ School of Pharmacy Hubei University of Science and Technology Xianning Hubei China

**Keywords:** colon cancer, cytokine, herpes simplex virus type 2, malignant ascites

## Abstract

**Background:**

Given malignant ascites with a terrible prognosis and a unique immune microenvironment, our purpose is to evaluate whether oncolytic herpes simplex virus type 2(OH2) is able to safely eliminate ascites of colon cancer and through which specific mechanism it exerts antitumor immunity.

**Methods:**

We established an ascites mice model through intraperitoneal injection of CT26 cells and obtained an appropriate dose range for in vivo tests. Efficacy and safety of OH2 were detected by weight of ascites, blood routine analysis, histopathological examination, and the survival time of mice. The specific mechanism underlying antitumor immunity was analyzed by cytometric bead array, flow cytometry, and single‐cell RNA sequencing. Furthermore, anti‐interleukin (IL)‐6R antibody tocilizumab was synchronously or sequentially delivered with OH2 to explore the role of the regional cytokine storm, mainly IL‐6 hypersecretion.

**Results:**

OH2 was able to eliminate ascites and significantly prolong the survival of mice‐bearing CT26 tumor cells by intraperitoneal injection, without obvious systemic damage to the main organs even though a regional cytokine storm. Hypersecretion of pro‐inflammatory cytokines, mainly IL‐6, and increased infiltration of CD4+ and CD8+ T cells were observed in ascites mice treated by OH2, compared with those treated by 5‐fluorouracil or nonresponders. Furthermore, the initial‐stage blocking of the IL‐6 pathway was able to considerably suppress antitumor immune responses driven by OH2. Surprisingly, we discovered upregulations of the immune checkpoint genes such as Cd274 and Pdcd1 by single‐cell RNA sequencing.

**Conclusions:**

OH2 could safely eliminate malignant ascites of colon cancer and convert the cold immune microenvironment by inducing a remarkably regional cytokine storm in ascites, mainly IL‐6, in the early stage of antitumor immune responses beyond directed oncolytic virotherapy.

## Graphical abstract

OH2 induced a remarkably regional cytokine storm through the direct and exhaustive oncolytic cytotoxicity. The immune checkpoint genes such as Cd274 (encoding PD‐L1) and Pdcd1 (encoding PD1) were up‐regulated of in OH2 group. The initial‐stage blocking to IL‐6 pathway was able to considerably suppress anti‐tumor immune responses driven from OH2.

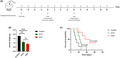

## 1 INTRODUCTION

Malignant ascites (MA) is a terrifying complication characterized by the accumulation of fluid in the abdominal cavity, and it is related to poor prognosis, especially advanced gastric cancer and colorectal cancer.^1^, ^2^ The primary treatments of malignant ascites are various therapies targeting malignant tumor cells such as chemotherapy and targeted therapy.^3^, ^4^, ^5^ For malignant ascites caused by gastrointestinal tumors, systemic or local infusion of chemotherapy alone is a common upfront line treatment which could also combine with other antiangiogenic agents, including vascular endothelial growth factor (VEGF) antagonists and immune checkpoint inhibitors for selected patients, confirmed by abundant preclinical and clinical evidences.^6^, ^7^, ^8^, ^9^ In addition, hyperthermic intraperitoneal chemotherapy (HIPEC),^10^ based on intraperitoneal chemotherapy that usually follows with cytoreductive surgery, has been shown to be a promising treatment to offer a palliative improvement for patients with metastatic colorectal cancer and peritoneal carcinomatosis. Despite the current advances mentioned above, there are still various shortcomings and limitations. First, therapies cannot fully halt the production of ascitic fluid which is mediated by peritoneal angiogenesis and augmented vascular permeability, mainly resulting from primary and secondary resistance of tumor cells to agents. Second, patients with poor performance status, especially elderly subpopulations, are not suitable for either chemotherapy or surgery, yet only able to receive palliative volume to relieve compression symptoms, which includes drainage of ascites by puncture, a peritoneal shunt. Unfortunately, it always leads to protein loss, electrolyte disorder, and even life‐threatening infection.

Oncolytic viruses (OVs) are a novel and promising therapeutic approach through two main mechanisms of action.^11^, ^12^, ^13^, ^14^ First, OVs can selectively replicate within tumors and directly induce cellular lysis in that tumors have sluggish or defective pathogen‐associated molecular pattern (PAMP) and damage‐associated molecular pattern (DAMP) responses, which make them particularly susceptible to virus infections. Second, antitumor immunity elicited by the OVs is the consequence of improved antigen cross‐priming and recruitment of immune cells into the tumor microenvironment (TME). Likewise, OVs infection in murine melanoma cells rapidly induced pro‐inflammatory cytokines, including IL‐6, type I IFN, and TNF‐α, which are not only immunostimulatory in function but also able to kill residual uninfected cancer cells.^15^, ^16^, ^17^, ^18^ Furthermore, the tumor stroma contributes to tumor growth and treatment resistance by acting as a physical barrier against effective drug delivery, including tumor‐targeting agents and even local injection of viruses. Thus, as an attractive stromal cell target, the fibroblast activation protein (FAP)^19^ is largely focused on at present. Moreover, it has been reported that oncolytic measles virus coeliac is administrated in patients of recurrent ovarian cancer, supported by promising results from preclinical studies and clinical trials.^20^, ^21^

Although recent studies have shown that immune checkpoint inhibitors (ICIs) can effectively induce antitumor immune responses in metastatic colorectal cancer (mCRC) with deficient mismatch repair (dMMR) and have been approved by the Food and Drug Administration (FDA), only <5% of tumor patients display dMMR. Unfortunately, a minority of patients (20%–30%) among this subtype still did not respond to immunotherapy alone.^22^ One strategy to overcome primary resistance to ICIs is to combine with OVs immunotherapy, chemotherapy, and/or targeted treatment.^23^

Oncolytic herpes simplex virus type 2 (also called OH2) first developed by our team is a novel oncolytic herpes simplex virus armed with GM‐CSF, with deletions in ICP34.5 and ICP47.^24^ Our OH2 is considered to be more potent in destroying tumors and spread more rapidly via syncytia formation than the other viruses, like adenovirus, measles virus, and herpes simplex virus type 1 (HSV‐1).^25^ OH2 was well tolerated and demonstrated durable antitumor activity in patients with metastatic esophageal and rectal cancer in the ongoing phase I/II clinical trial.^26^

Undoubtedly, based on the above mechanism and reasonable speculation, we are highly interested in exploring the efficacy and safety of our OH2, by treating malignant ascites in a mouse model of colorectal cancer, converting the cold immune microenvironment to hot, and ultimately, generating a reasonable platform to incorporate ICIs to activate antitumor immunity.

## 2 MATERIALS AND METHODS

### 2.1 Cell culture

The CT26 mouse colon cancer cells were purchased from the Cell Resource Center of Peking Union Medical College and were cultured in DMEM (Dulbecco’s modified Eagle’s media)/F12 medium supplemented with 10% NBS, 100 μg/ml of streptomycin, and 100 U/ml of penicillin and incubated at 37°C with 5% CO_2_ and saturated humidity. Vero cells (from ATCC) were maintained in DMEM supplemented with 10% FBS containing 2 mmol/L l‐glutamine, 100 U/ml penicillin, and 100 μg/ml streptomycin (Gibco) and grown at 37°C under humidified conditions.

### 2.2 Viruses

OH2 (HG52/34.5‐/47‐hGM‐CSF) used in this research was produced according to the standard protocol.^24^ The production batch number of the OH2 virus was VBT‐oHSV2‐hGM‐CSF‐201908A‐KZH‐NS.

### 2.3 Oncolytic ability of OH2 in vitro

The viral multiplicity of infection (MOI) of OH2 to CT26 tumor cells was tested by the MTT experiments, and compared with fluorouracil (5‐FU, as a positive control).

Colon cancer cells (CT26) were seeded at 1 × 10^4^ cells per well. Cell proliferation was determined by MTT assay on days 0, 2, and 4 of cell culture. The absorbance of 100 μl of supernatant was measured at 490 nm with a reference wavelength of 630 nm by using a microplate reader (Biotek ELx800). Background absorbance from the control wells, which contained the culture medium but without cells, was subtracted.

### 2.4 Mouse experiments

Six‐week‐old BALB/c mice, purchased from the Hubei Food and Drug Safety Evaluation Center, were housed under specific pathogen‐free conditions. The general health status of the mice was monitored daily.

### 2.5 In vivo activity evaluation of OH2 in a CT‐26 mouse model

CT26 tumor models were established by subcutaneous injection of 1 × 10^6^ CT26 cells on the right flank of BALB/c mice. When the tumor volume reached about 100 mm^3^, mice were randomly divided into five groups (each group contained six mice, a total of 30 mice), and three different doses of OH2 (1E6, 1E5, and 1E4 CCID50) were injected into the tumor, respectively. The control groups were treated with 5 mg/ml of 5‐fluorouracil (positive control group) and serum‐free medium (negative control group). The injection volume was 100 μl, and mice were injected subcutaneously once every 3 days for three consecutive treatments on the 0th, 3rd, and 6th day. The tumor volumes in each group were monitored for a period over 21 days by measuring the size of long (L) and short (W) diameters of tumors with vernier calipers every 3 days, to generate a tumor volume growth curve. After 21‐day tumor implantation, most of the mice were sacrificed by anesthesia, and then the tumor masses, and serums were collected for further analysis.

### 2.6 Mouse malignant ascites model establishing

BALB/c mice were injected intraperitoneally (1 × 10^6^ CT26 cells per mouse, 100 μl). After 4 days, the tumor‐bearing mice were then randomly divided into three groups (each group contained 18 mice), injected intraperitoneally with OH2 virus (1 × 10^6^ CCID50 per mouse, 250 μl), 5‐fluorouracil (positive control group), or serum‐free medium (negative control group) on 0th, 4th, and 8th days for three times. After 4‐ and 9‐day tumor implantation, most of the mice were sacrificed by anesthesia, and then the serums and ascites were collected for further analysis. Six mice were selected by random sampling to continuously be raised and followed up for survival time.

### 2.7 Ascites collection and pathological sample preparation

On the 9th day after the first administration, six mice from each group were sacrificed (and the remaining six mice continue to be treated for survival analysis). Immediately after the animals were sacrificed, the ascites weights (g) were measured. The cellular components were collected from ascites for flow cytometric analysis and single‐cell RNA sequencing, and the cell‐free ascites fluid was stored at −70°C for cytokines detection.

In addition, main mice organs (three random mice per group) were carefully excised and stored overnight in freshly prepared 4% formaldehyde solution for pathological examination. Hematoxylin and eosin staining were performed on various organs and tissues (brain, spinal cord, thymus, lung, heart, esophagus, stomach, small intestine, colon, rectum, mesenteric lymph nodes, liver, pancreas, spleen, kidney, adrenal gland, perirenal ganglion, bladder, etc.), and peripheral blood samples were obtained for biochemical detection to assess the toxicity of OH2.

### 2.8 Cytokine detection

The presence of cytokines in blood plasma and ascites supernatant was determined by the CBA assay (Cytometric Bead Array, BD Biosciences, San Jose, CA, USA). The kit used for the analysis of cytokines was Mouse Th1/Th2/Th17 Cytokine Kit.

### 2.9 IL‐6 signaling blocked by tocilizumab

The mouse colon cancer cells (CT26) were injected intraperitoneally (1 × 10^6^ CT26 cells per mouse, 100 μl) as described above. After 4 days, the tumor‐bearing mice were randomly divided into four groups (each group contained 12 mice, a total of 48 mice), injected with OH2 virus (1 × 10^6^ CCID50 per mouse, 250 μl), and added tocilizumab (named Actemra from Roche, a monoclonal anti‐IL6R neutralizing antibody) by 10 mg/kg IP once. Some mice were inspected every 3 days, and killed on day 9, and others continued to be raised and followed up for survival time.

### 2.10 Flow cytometric analysis

Flow cytometric analysis was performed with a FACS Canto II instrument (BD Bioscience) to collect 500 μl immune‐stained cells under no more than 2500 target populations/sec and data were processed using the FlowJo 7.6.1 software.

### 2.11 Single‐cell RNA sequencing (scRNA‐seq) and data analysis

Single‐cell capture and library preparation were performed by the BD Rhapsody Single‐Cell Analysis System (BD Biosciences). The libraries were quantified using the Agilent 2100 Bioanalyzer (Agilent) and the Qubit 4.0 (Thermo Fisher Scientific) and were sequenced on Illumina NovaSeq 6000 (Illumina) with 300‐bp reads (150‐bp paired‐end reads).

Raw reads produced by the Illumina pipeline in FASTQ Format were preprocessed to obtain clean reads for further analysis. BD Rhapsody™ WTA Pipeline was used with clean data to perform quality control, extract barcodes and UMI, and align to the mouse reference genome (GRCm38‐PhiX‐gencodevM19). A k‐nearest neighbor graph was executed with Euclidean distances in the space of the first 10 significant principal components, and the clustering results were visualized by tSNE and UMAP, respectively. The Marker genes were identified with the default parameters by Find All Markers in Seurat. The original clusters were annotated with the data set of Mouse RNAseq Data by SingleR (v.1.0.1). Based on annotated clusters, the T cell cluster was extracted by subset function for subanalysis.

### 2.12 Statistical analysis

Statistics were performed with SPSS version 23.0. Data were presented as the mean ± SEM. Kaplan–Meier test was used to analyze the survival of mice. One‐way ANOVA (for multiple comparisons) or two‐tailed Student’s *t*‐test (two groups) was used to test the differences in ascites weights and cytokines among multiple groups. A value of *p* < 0.05 was considered statistically significant.

### 2.13 Ethical statement

All animal tissue collection procedures were performed according to protocols approved by the Biological Studies Animal Care and Use Committee, Hubei Province, PR China.

## 3 RESULTS

### 3.1 Oncolytic ability of OH2 in vitro and in vivo

The viral inhibition MOI was determined by MTT assay on days 0, 2, and 4 of cell culture.

We discovered that OH2 had a strong oncolytic ability in infecting cells with MOI = 1.0, 5.0, and 10.0 for 96 h and could significantly lyse malignant cells. The inhibition peak appeared at 48 h among all MOI, but the lysis function was slightly increased in the later period (Figure S1A,B). Furthermore, the oncolytic ability was not absolutely concentration‐dependent, as we found that the oncolytic activity has already reached a platform at MOI = 5.0, despite obviously higher than MOI = 1.0, even if at MOI = 10.0 dose of viruses, no more than 5% decrease of cell viability was achieved. Therefore, the rational concentration range used by us should be closed to MOI = 5.0 for the next in vivo experiment. Similarly, administration schedules for mice were intervened three times by a 4‐day interval.

**FIGURE 1:**
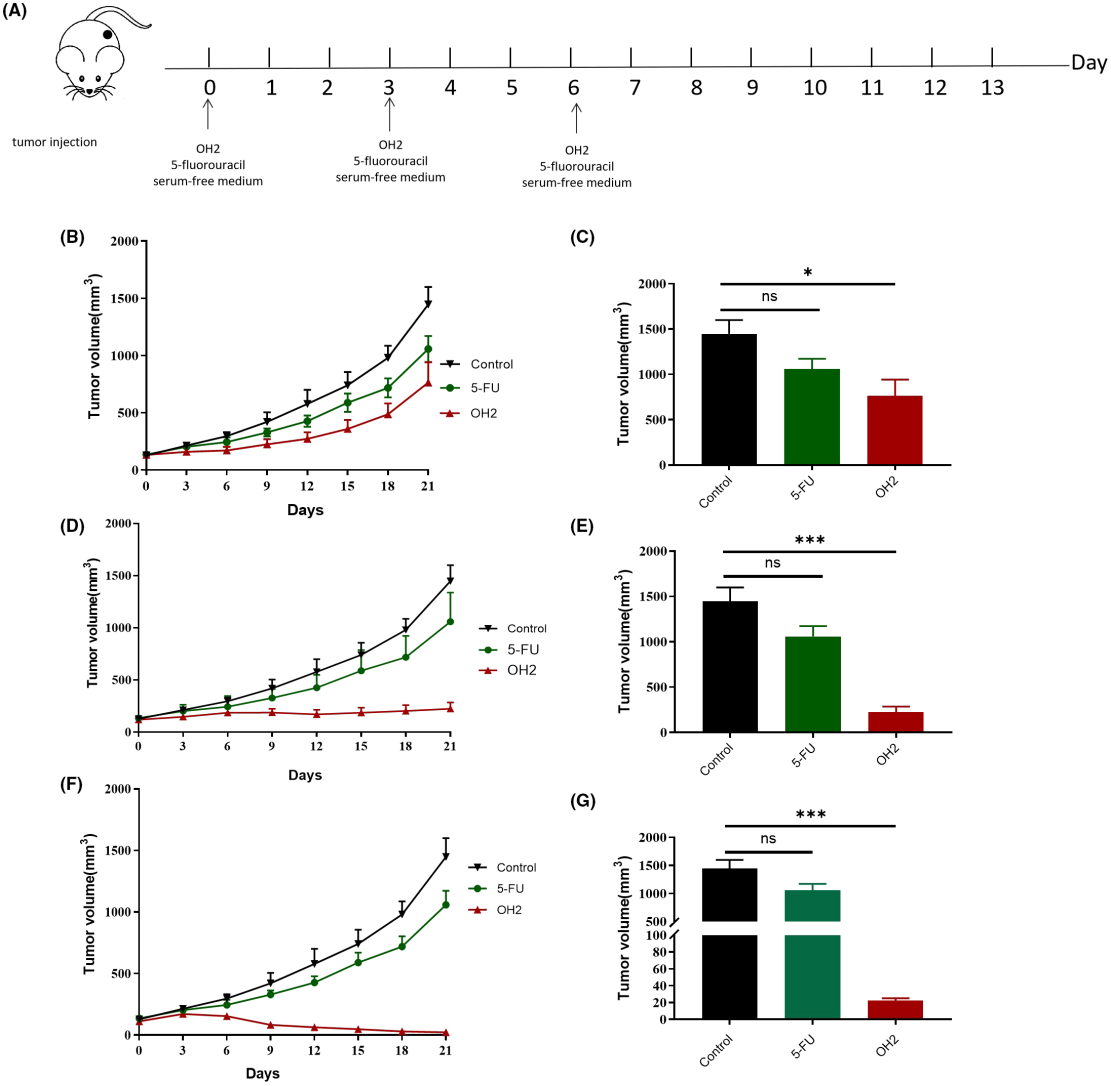
OH2 inhibited tumor growth from first administration to day 21 in vivo. (A) The schema of treatment protocol; (B, C) tumor volumes on day 21 and growth curves in group E4; (D, E) tumor volumes on day 21 and growth curves in group E5; (F, G) tumor volumes on day 21 and growth curves in group E6. Tumor volume: mean ± SEM; one‐way ANOVA or Student’s *t*‐test (*p* > 0.05, ns; *p < 0.05; **p < 0.01; ***p < 0.001)

To assess the efficacy and safety of OH2 in the treatment of colon cancer, we performed a dose‐escalation study in the mice model (six mice per group) to confirm whether OH2 had a strong capability to induce direct oncolysis and antitumor immune response. We found that OH2 showed activity against tumors in a dose‐dependent manner (Figure 1). The significant antitumor effect in E6 dose groups showed that the tumors were greatly reduced by OH2 (tumor volume was 22.40 ± 3.04 mm^3^, compared with 1058.41 ± 151.97 mm^3^ in the 5‐FU group and 1447.83 ± 113.85 mm^3^ in negative controls, respectively). In the E5 dose group, tumor volumes of the OH2 group (mean tumor volume was 224.01 ± 63.66 mm^3^) were also smaller than those of the 5‐FU group on day 21 after the first treatment. In the E4 dose group, OH2 (mean tumor volume was 764.99 ± 177.46 mm^3^) exerted a certain antitumor effect that was a bit stronger than 5‐FU. In terms of safety, no death in 48 mice was observed and the general body condition of the animal remained unchanged throughout the observation period.

In summary, our outcomes showed that OH2 had promising clinical value, and E6 and probably E5 were suitable dosages for further ascites research.

### 3.2 OH2 inhibits ascites production in mouse

We found the CT26 ascites model was successfully established while CT26 tumor‐bearing mice exhibited a sharp increase in abdominal circumference on day 4, which was confirmed by dissected preexperiment mice.

**FIGURE 2:**
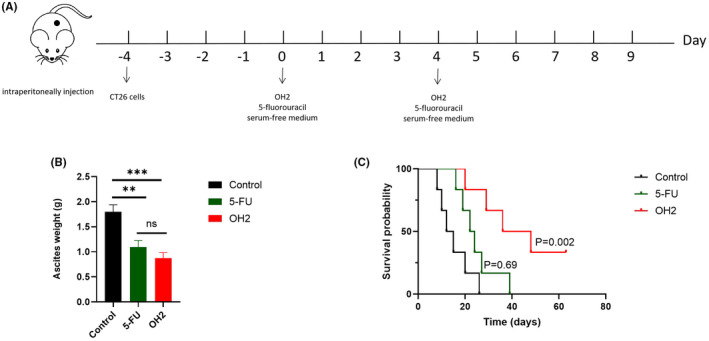
OH2 inhibited ascites production and prolonged survival time. (A) The schema of treatment protocol; (B) OH2 inhibited ascites production in mouse ascites weight on day 9 since the first treatment; (C) Kaplan–Meier curves of overall survival time in different groups. Ascites weight: mean ± SEM; one‐way ANOVA (*p* > 0.05, ns; *p < 0.05; **p < 0.01; ***p < 0.001)

The average weight of ascites on day 9 since the first treatment was shown (Figure 2B). The ascites weight of the OH2 treatment group was significantly lower than that of the control group (0.91 ± 0.12 g, 1.80 ± 0.14 g), while fluorouracil had mild efficacy for ascites (1.10 ± 0.13 g). However, there was no statistical difference between the OH2 and fluorouracil groups.

### 3.3 OH2 prolonged survival of mice with ascites

In addition to local therapeutic effects, such as symptom improvement and increased vitality of mice, we found that the survival time of the mice in the OH2 group was significantly longer than that in the other two groups. The median survival time was 36 (95%CI: 13.20 to 58.81), 22 (95%CI: 16.00–28.00), and 12 (95%CI, 6.00–18.00) days in OH2 group (*n* = 6), fluorouracil (*n* = 6), and control groups (*n* = 6), respectively (Figure 2C). Of note, compared with the blank control group, fluorouracil could slightly improve survival time.

### 3.4 Levels of cytokines after OH2 infection in ascites

While oncolytic viruses like T‐VEC® have been approved for cancer immunotherapies partially owing to their potential to awaken the immune system, the immune regulations remain unclear in malignant ascites. Moreover, our previous studies have found that oncolytic viruses could activate T cells and NK cells, which may be the result of a large number of secretion of cytokines (data unpublished). Therefore, we first conducted cytokine assays to examine the influence of our OH2 on the immune systems.

**FIGURE 3:**
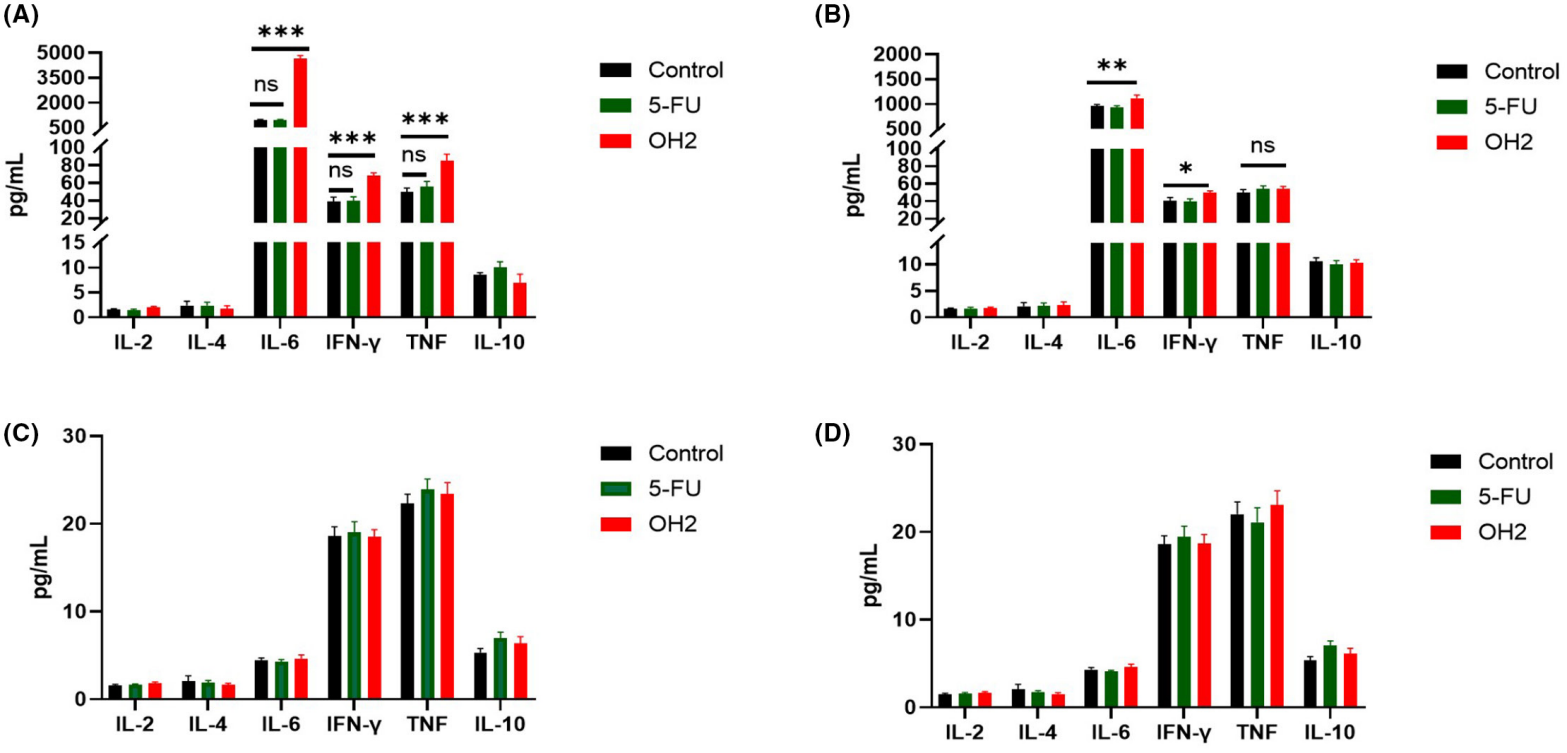
The levels of six cytokines between the OH2 group and the other control group from ascites and serums. (A) the cytokine levels of ascites on day 4 since the first treatment; (B) the cytokine levels of ascites on day 9 since the first treatment; (C) the cytokine levels of serums on day 4 since the first treatment; (D) the cytokine levels of serums on day 9 since the first treatment

We found that the levels of multiple cytokines, especially for IL‐6 and IFN‐γ, in ascites on day 4 since the first treatment were higher (more than four times) in the OH2 group (*n* = 6) than those in the control group (*n* = 6). The levels of TNF and IFN‐γ also showed an upward trend (Figure 3). Moreover, at the end of the experiment (day 9), the cytokines in the mice that still had ascites decreased significantly, although they were slightly higher than in controls. Importantly, the mouse peripheral blood cytokine levels were substantially normal or difficult to be detected, like an intratumoral injection of mice mentioned above. The results suggested that OH2 might initially promote inflammatory forms of cell death through its direct oncolytic cytotoxicity characterized by faster and stronger hypersecretion of pro‐inflammatory cytokines, mainly IL‐6. Therefore, whether and how the regional cytokine storm subsequently activated the immune system and collaboratively played an antitumor role was our further purpose to investigate.

### 3.5 OH2 activates CD8+ T and CD4+ T cells in ascites

It was important to investigate the activation of immune cells to dramatically alter the immune microenvironment of ascites. Furthermore, our previous studies in vitro have found that OH2 could promote the proliferation of various lymphocytes when cocultured with peripheral blood lymphocytes.^24^

**FIGURE 4:**
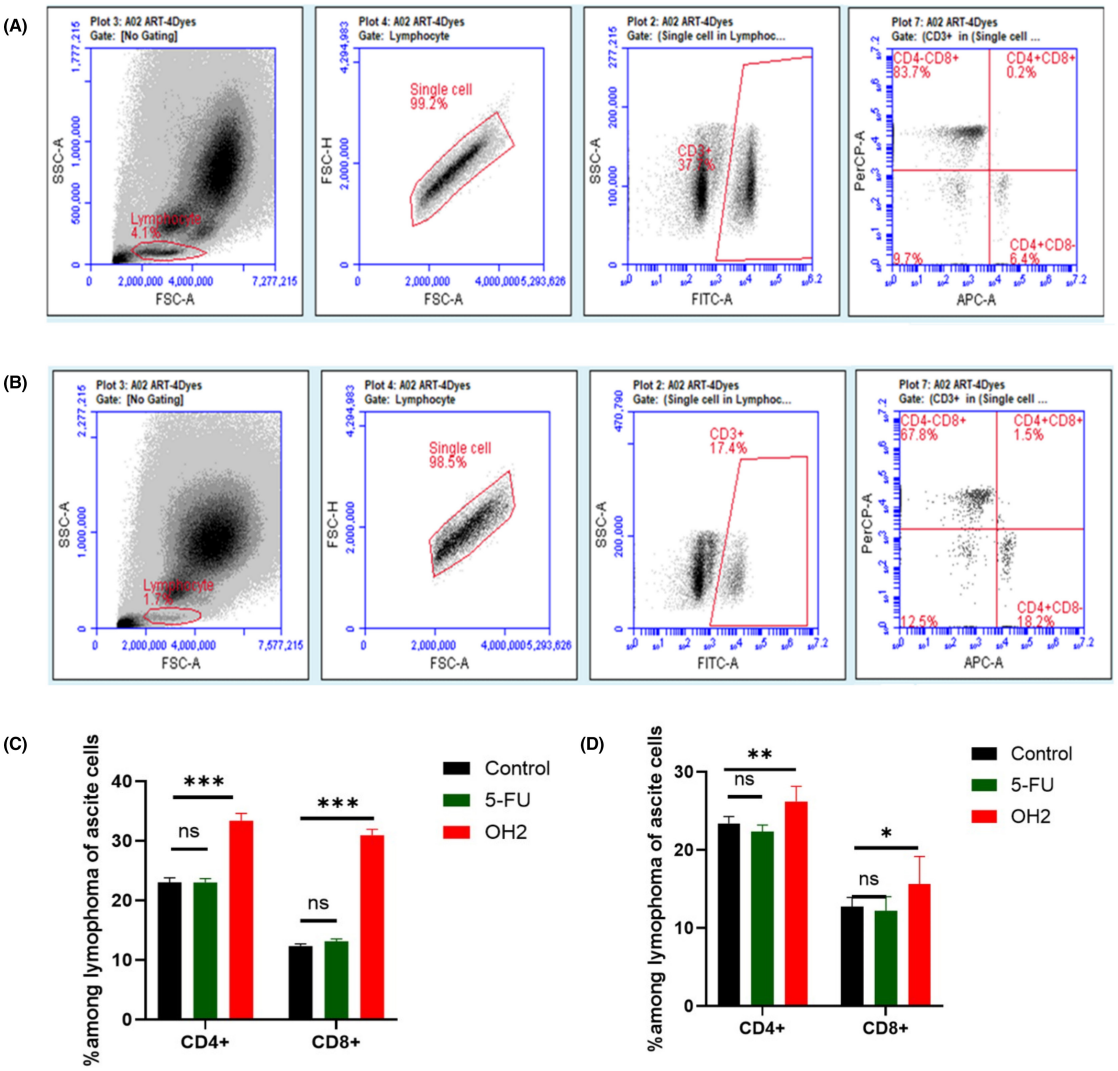
OH2 increased numbers of CD4+ and CD8+ T cells in murine ascites on day 4 and day 9 by flow cytometric analysis. (A) Representative results; numbers were percentages. CD8+ and CD4+ T cells in ascites from control; (B) Representative results; numbers were percentages. CD8+ and CD4+ T cells in ascites from OH2 treatment; (C) Percentages of CD4+ and CD8+ T cells in murine ascites on day 4; (D) Percentages of CD4+ and CD8+ T cells in murine ascites on day 9

Through flow cytometric analysis that employed cell surface marker staining, our data revealed that there were an increased number of CD4+ T cells and CD8+ T cells in ascites at an early stage of infection (day 4) by OH2(*n* = 12) compared with PBS‐ (*n* = 6) or fluorouracil (*n* = 6)‐treated tumors. We thought increased numbers of CD4+ and CD8+ T cells have resulted from the novel chemotactic influx stimulated by the cytokines, although it was not known whether the proliferation of CD4+ T cells was also involved (Figure 4). Moreover, at the end of the experiment (day 9), the number of CD4+ T cells and CD8+ T cells maintained general stability, but their phenotype or/and function status were unclear, which also became our aim to explore.

### 3.6 T cell subpopulations and functional analysis by scRNA‐seq

**FIGURE 5:**
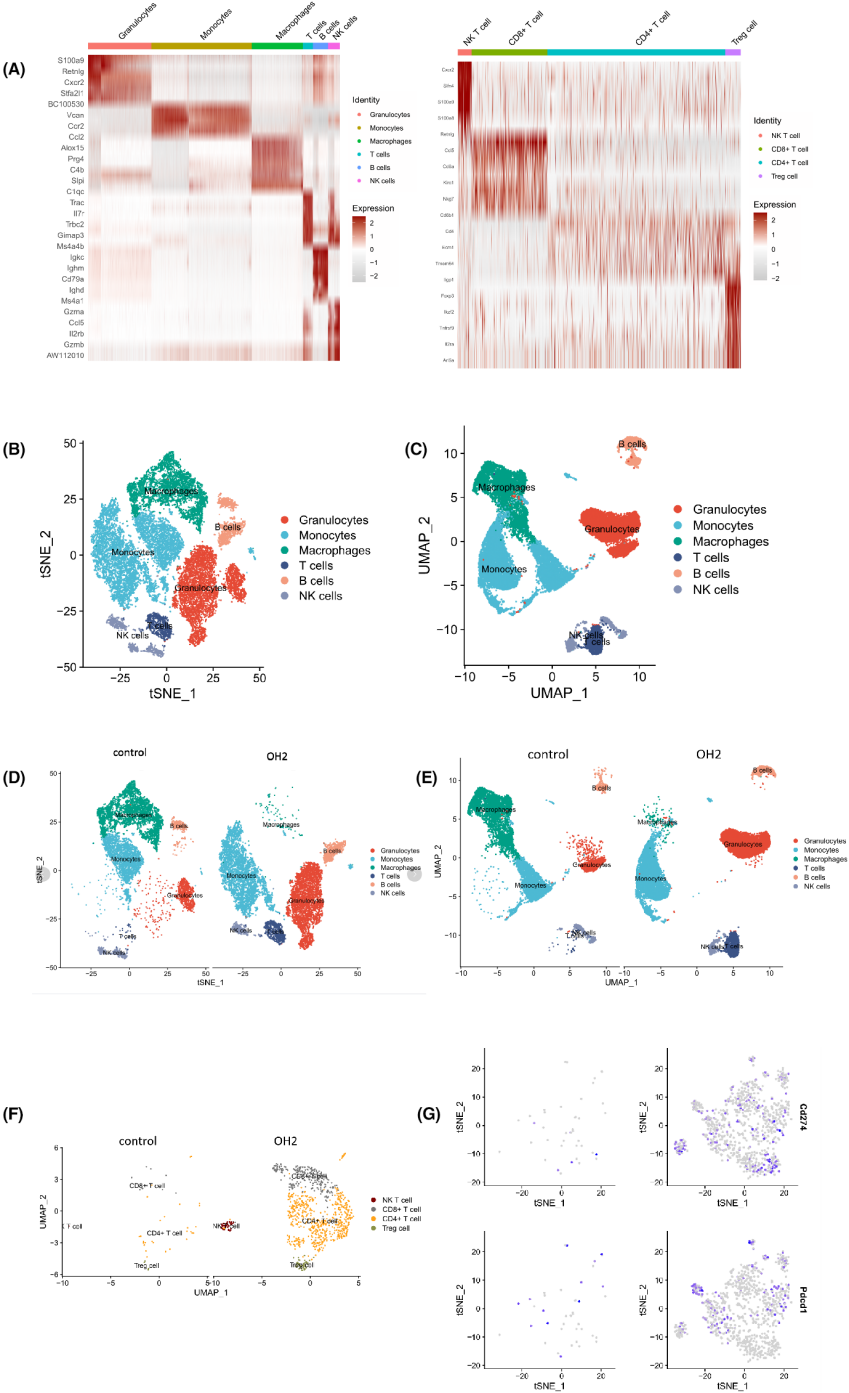
Characterization of T cell subtypes stimulated by Oncolytic OH2 in ascites. (A) Based on the marker genes, we defined each cell group. Red showed for high expression. (B) Each of the T cell clusters was identified by marker genes. Red showed high expression from 15,139 cells and 15,546 cells in the control and OH2 groups, respectively. (C, D) Based on the marker genes, we defined a total of six cell groups through tSNE and UMAP. (E, F) Histogram tSNE and UMAP showing T cells group (dark blue) were stronger upregulated in OH2 compared with the control group. (G) Orange and black plots show both CD4^+^ and CD8^+^ T cells involved in OH2 and control groups, respectively. (H) Representative gene expressions of Cd274 and Pdcd1 in T cell were stronger upregulated in OH2 compared with the control group

Next, we used unsupervised clustering data analysis to separate the T cells into distinct groups of immune populations (Figure 5) by scRNA sequencing based on the expression of known markers for each population. We found that among the six clusters of cells (Figure 5C), the proportions of T cells, monocytes, macrophages, B cells, NK cells, and neutrophils sharply changed along the OH2 infection. Among the upregulated T cells, further analysis showed that both CD4+ and CD8+ T cells were increased significantly, while the effect on the former was stronger (Figure 5E,F). The massive activation of neutrophils may be related to the release of cytokines, one of the reasons for the local cytokine storm. Furthermore, the proportion of NK cells mildly increased since OH2 with the upregulation of IFN‐γ, a gene representing the immune stimulation activity (Figure 5C). Notably, the immune suppressive Treg cells also were enriched in ascites of OH2 treatment, suggesting that the regulation of OH2 to the immune microenvironment is not a direct yin and yang dichotomous effect but an interactive influence. Meanwhile, the expression level of genes involved in immunosuppression elevated along with the increase of T cell proportion. For instance, we found the upregulation of the immune checkpoint genes such as Cd274 (encoding PD‐L1) and Pdcd1 (encoding PD1) in the OH2 treatment group in T cells and neutrophils (Figure 5F,H).

### 3.7 Blocking the IL‐6 signaling pathway by tocilizumab downregulates OH2 activity to regress ascites

We hypothesized that IL‐6 played dominant immunomodulatory signaling to increase T‐cell trafficking, as IL‐6 production was exceedingly high by about 20 times compared with other pro‐inflammatory factors. Moreover, defining thoroughly the nature of tumor cell death in ascites induced by the combination of IL‐6, TNF‐α, and IFN‐γ, which had previously been reported to induce immunologically lymphocyte proliferation, and dissecting the signaling pathways are critical to understand the interplay between mechanisms of cell death and how they may affect the tumor microenvironment.

**FIGURE 6:**
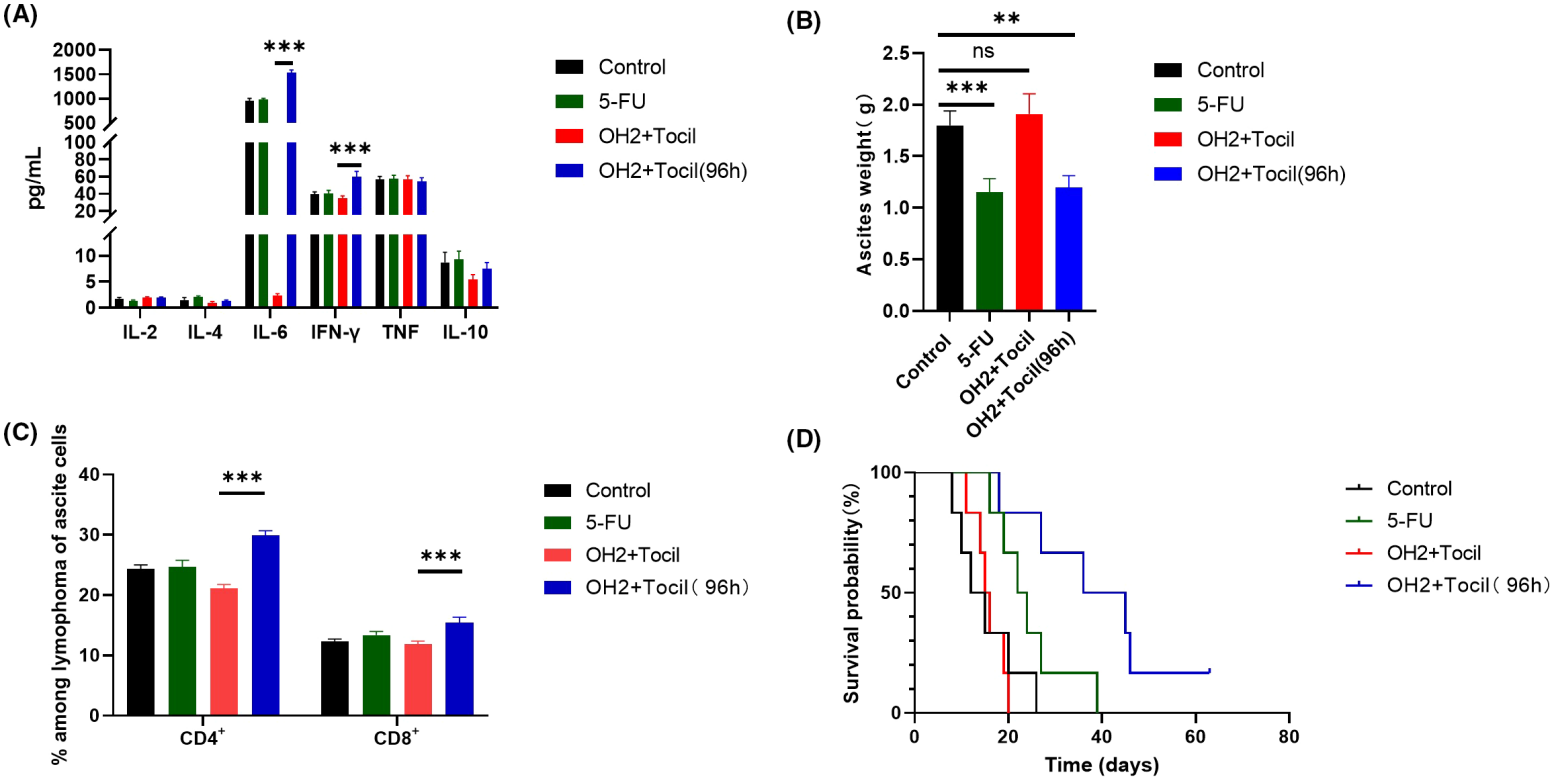
Blocking the IL‐6 signaling pathway by tocilizumab‐downregulated OH2 activity. (A) The levels of six cytokines between the OH2 group and the other control groups (simultaneous tocilizumab exposure); (B) OH2 inhibited ascites production in mice; (C) OH2 increased numbers of CD4+ and CD8+ T cells in murine ascites on day 9 (simultaneous tocilizumab exposure); (D) Kaplan–Meier curve for overall survival

Next, we evaluated the efficacy of tocilizumab for OH2 inhibition of tumor ascites growth. We found delayed ascites growth driven by OH2 was significantly eliminated in the model animals through synchronous tocilizumab exposure (Figure 6) and failed to prolong survival, unlike in mouse models with undamaged IL‐6 signaling. However, the OH2 effects of ascites blockage cannot be offset when IL‐6 signaling was interfered by pharmacological inhibition with tocilizumab after virus administration of 96 h because of the ultimate initiation of potent anticancer immunity. Third, we analyzed levels of six cytokines and found that IL‐6 inactivation may also strongly downregulate TNF‐α and IFN‐γ release as well as other inflammatory compartments after OH2 infection. Notably, OH2 also cannot be sufficient to increase the numbers of CD4^+^ T cells and CD8^+^ T cells upon IL‐6 signaling inhibition, suggesting again that the inflammatory storm is the leading cause of immune activation.

These findings not only demonstrated that the IL‐6 signaling axis was an important driver of antitumor immune responses but also suggested that OH2, a candidate immunotherapeutic agent, could be particularly efficacious in disrupting complex communication events within the TME via rapid cytokine production.

### 3.8 Toxicity

Blood biochemical and blood routine analyses were performed to understand the long‐term biosafety of OH2, and no significant changes were observed. In addition, there were also no substantial changes in serum cytokines.

Vital organs, including esophagus, gastroesophageal junction, stomach, small intestine, colorectal, spleen, adrenal gland, ovary, fallopian tube, uterus, heart, liver, kidney, bladder, ureter, abdominal lymph node, pancreas, spinal cord, cerebellum, and brain, were harvested for histopathologic examination and no significant pathological changes were found in the organs of mice with malignant ascites treated by OH2 (Figure S2 **)**.

## 4 DISCUSSION

The tumor immune microenvironment is a sophisticated niche consisting of cancerous cells and various noncancerous components hindering the natural host immune responses and the efficacy of cancer immunotherapies together. A proposed approach to overcome the lack of the immune response is to remove coinhibitory signals through anti‐PDL‐1/PD1 and supply costimulatory signals through anti‐OX40 or anti‐GITR.^27^, ^28^, ^29^, ^30^ The concern with these approaches would be the concurrent increase in undesired side effects which surely require careful evaluation. Hence, a proposed approach that could in fact be effective and without obvious toxicities in the case of tumor ascites microenvironment is a particularly interesting research area.

To date, the route oncolytic viral therapies delivered in sufficient quantity of clinical efficacy is mainly via loco‐regional or direct inoculation,^31^, ^32^ for example, T‐VEC®. However, multiple shortcomings of OVs limit their clinical application. First, the risk of puncture complications would be unavoidable, especially for deeply seated solid tumors. Second, the rapid distribution of oncolytic viruses into the tumor is still difficult to succeed. Hence, intraperitoneal delivery of OVs is a smart choice with unique advantages in ascites treatment. Moreover, as we know, ascites represents the microenvironment without physical barriers, similar to that of CAR‐T application to treat blood malignant tumors, in which a certain number of tumor cells are quickly infected and surrounded by ubiquitous viruses. In addition, compared with systematic administration, intraperitoneal perfusion is equipped with a longer half‐life.

In our study, the mice model demonstrated that intraperitoneal perfusion of OH2 could markedly suppress ascites production and improve survival time with no obvious pathological damage in major organs. Notably, the effect of ascites treatment may be better than that of solid lesions by intratumoral injection, which was mostly lacked T‐cell trafficking and consequently, the establishment of a successful immune therapeutic efficacy.^33^

We thought that the “regional cytokine storm” from OH2 could induce the remodeling of the ascites immune microenvironment in which antitumor immune phenotype and functionality were established, especially effector T cells with upregulation of the immune checkpoint genes such as Cd274 and Pdcd1 (Figure. 6E,F). The reasons, we speculated, were that viruses in ascites could quickly infect a large number of tumor cells, like in vitro, and induce antitumor immunity, including both antiviral immunity and acquired resistance mechanisms, such as the upregulation of the immune checkpoint that has not been fully established. Thus, the net effect of OH2 is the reduction of ascites and local immune‐activated states. Of the cytokines, our study found that the high concentration of IL‐6 in ascites was related to the number of T cells, and after early stage rather late‐stage blocking, the therapeutic effect was significantly reduced. Previous studies have shown that IL6 can bind to IL6R and signal through JAK‐STAT to lead to a broad range of effects in both the hematopoietic and nonhematopoietic compartments. In the immune compartment, IL‐6 has a profound impact on increasing CD8^+^ T‐cell trafficking, cytotoxic CD8^+^ T‐cell differentiation and antibody production, and decreasing regulatory T cell differentiation, while tumor cells can also express and/or respond to IL‐6, contributing to increased proliferation and invasion.^34^, ^35^ Moreover, upregulation of pro‐inflammatory TNF and IFN‐γ in the ascites of mice caused by OH2 has also been recognized, as important markers associated with sensitivity to immunostimulatory agents.^36^, ^37^ We believed that these cytokines may act to either activate immune response or synergistically, cooperate with IL‐6 which may be a stronger activating signal, although we have not performed inhibition experiments to confirm.

Overall, the present study shows that the speed to trigger regional cytokine storm is a very important factor for OH2 to eliminate malignant ascites. Second, to the best of our knowledge, our data first demonstrate the novel OH2 has a key oncolytic potency in ascites to induce super‐reactive inflammatory cytokine releasing to convert the regional cold immune microenvironment to hot^38^, ^39^, ^40^ and possibly upregulate immune checkpoint expressions. At last, the next generation of OH2 armed by PD‐1 antibody and bi‐ and tri‐specific T cell engager antibody (targeting FAP and/or CD3 molecules) to further improve the antitumor effect alone and/or combining with other unimmunotherapies or immunotherapies also require further development.

## CONFLICT OF INTEREST

The authors declare no competing interests.

## AUTHORS’ CONTRIBUTIONS

Conceived and designed the experiments: SD, BlL, SH, and FxZ. Performed the experiments: SD, FG, YZ, QC, and SqZ. Analyzed the data: SD, Yu Qian, JW, SH, and FxZ. Wrote the paper: SD, SH, and FxZ.

## INSTITUTIONAL REVIEW BOARD STATEMENT

The animal study protocol was approved by the Animal Ethics Committee of Hubei University of Technology (HBUT No. 2018010).

## Supporting information


Figure S1

Figure S2

## Data Availability

The data that support the findings of this study are available from the corresponding author upon reasonable request.
