# Supplementary figures and images for: A novel oncolytic virus induces a regional cytokine storm and safely eliminates malignant ascites of colon cancer
Source: Cancer Med. 2022 May 5;11(22):4297–309. doi: 10.1002/cam4.4772 (PMC9678102; doi:10.1002/cam4.4772)

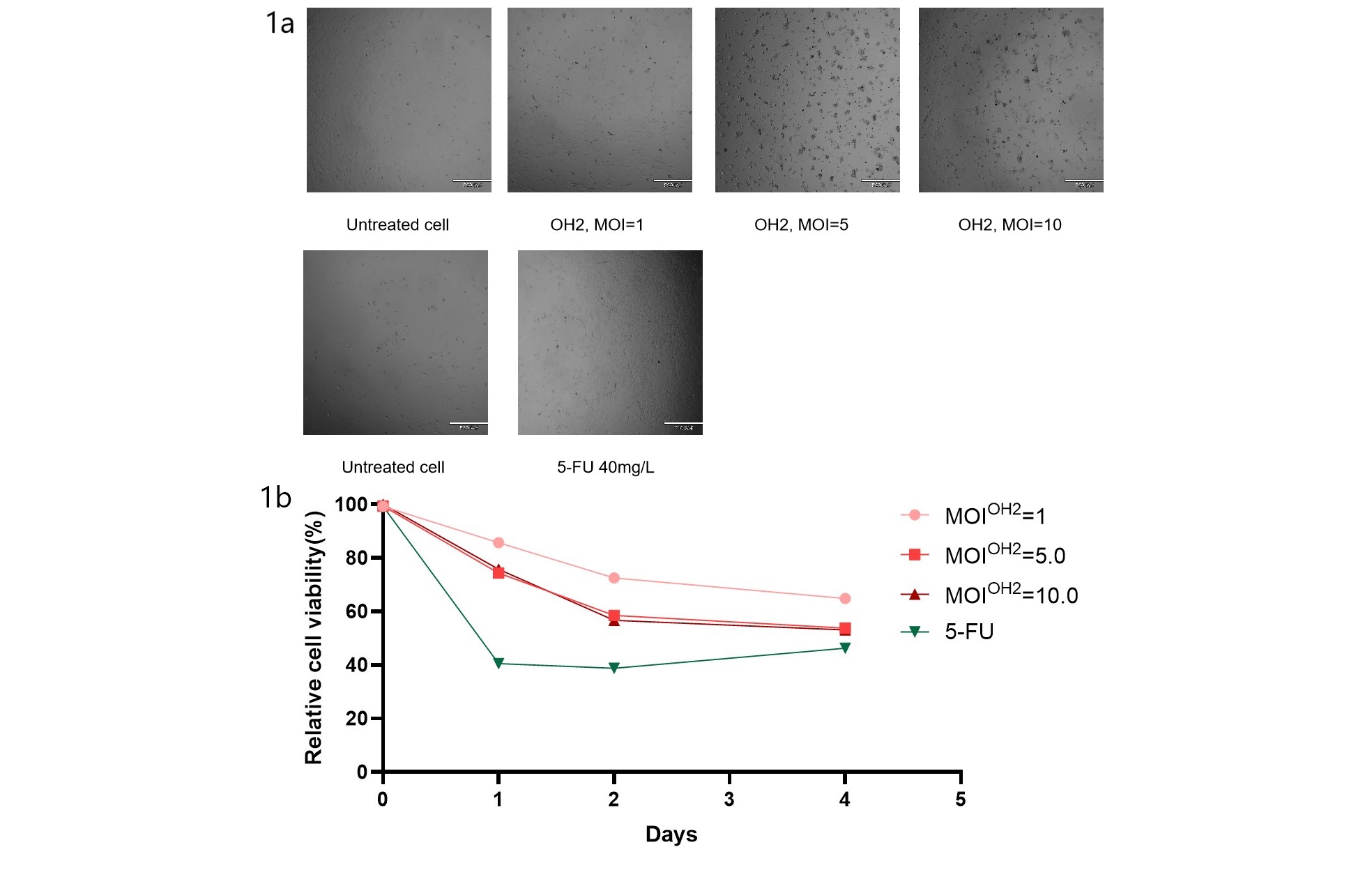

Supplement: Supplementary file 1 — Figure S1 [file CAM4-11-4297-s001.jpg]

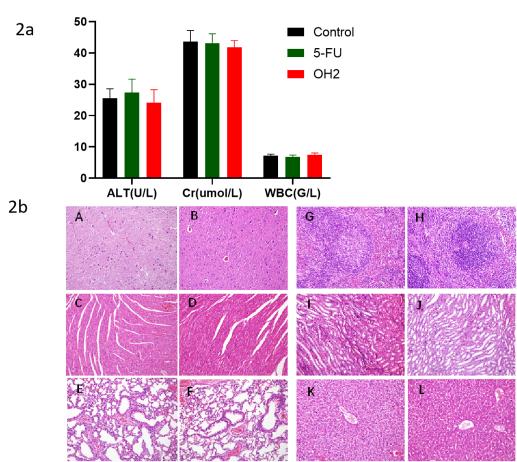

Supplement: Supplementary file 2 — Figure S2 [file CAM4-11-4297-s002.jpg]
